# Seeing the forest for the trees: Assessing genetic offset predictions from gradient forest

**DOI:** 10.1111/eva.13354

**Published:** 2022-02-25

**Authors:** Áki Jarl Láruson, Matthew C. Fitzpatrick, Stephen R. Keller, Benjamin C. Haller, Katie E. Lotterhos

**Affiliations:** ^1^ 5922 Department of Natural Resources Cornell University Ithaca New York USA; ^2^ 14725 Appalachian Laboratory University of Maryland Center for Environmental Science Frostburg Maryland USA; ^3^ Department of Plant Biology University of Vermont Burlington Vermont USA; ^4^ 5922 Department of Computational Biology Cornell University Ithaca New York USA; ^5^ Department of Marine and Environmental Sciences Northeastern University Marine Science Center Nahant Massachusetts USA

**Keywords:** biodiversity, climate change, gradient forest, landscape genetics, local adaptation, population genetics, quantitative genetics, simulation, SLiM

## Abstract

Gradient Forest (GF) is a machine learning algorithm designed to analyze spatial patterns of biodiversity as a function of environmental gradients. An offset measure between the GF‐predicted environmental association of adapted alleles and a new environment (GF Offset) is increasingly being used to predict the loss of environmentally adapted alleles under rapid environmental change, but remains mostly untested for this purpose. Here, we explore the robustness of GF Offset to assumption violations, and its relationship to measures of fitness, using SLiM simulations with explicit genome architecture and a spatial metapopulation. We evaluate measures of GF Offset in: (1) a neutral model with no environmental adaptation; (2) a monogenic “population genetic” model with a single environmentally adapted locus; and (3) a polygenic “quantitative genetic” model with two adaptive traits, each adapting to a different environment. We found GF Offset to be broadly correlated with fitness offsets under both single locus and polygenic architectures. However, neutral demography, genomic architecture, and the nature of the adaptive environment can all confound relationships between GF Offset and fitness. GF Offset is a promising tool, but it is important to understand its limitations and underlying assumptions, especially when used in the context of predicting maladaptation.

## 1 INTRODUCTION

There is an urgent societal need to better predict how specific genotypes perform in different environments. For example, in order for assisted gene flow to contribute to robust populations of harvested forests (Aitken & Bemmels, 2016) or key fish habitat reef systems (Matz et al., 2020), transplanted genotypes must be good candidates to increase the overall population fitness. Similarly, restoration of degraded ecosystems depends heavily on the identification of optimally adapted source populations if restoration efforts are to be successful (Houde et al., 2015). Likewise, climate change is a growing threat to biodiversity (Nunez et al., 2019; Urban et al., 2016) and there is a need to address environmental impacts on vulnerable populations. Most efforts to assess climate change impacts use species‐level distribution models (Aitken et al., 2008; Ellis et al., 2012; Pacifici et al., 2015), but genomic data are increasingly being incorporated to provide population‐level assessments (Hoban et al., 2016; Rellstab et al., 2015; Waldvogel, Feldmeyer, et al., 2020). For all these applications, predictive models can provide a powerful means to inform conservation (Bland et al., 2015; Freer et al., 2018; Razgour et al., 2018).

One particular *machine learning* algorithm that has increasingly been used to quantify and predict changes in the composition of biodiversity is Gradient Forest (GF) (Bay et al., 2018; Capblancq et al., 2020; Fitzpatrick & Keller, 2015; Layton et al., 2021; Ruegg et al., 2018). GF was conceived to characterize changes in community composition (Ellis et al., 2012), but more recently GF has been used to identify environmental drivers of allele frequency variation, as well as to forecast the degree of potential maladaptation of locally adapted populations under new environments (Fitzpatrick & Keller, 2015). While GF is growing in use as a predictive tool to identify potential environmentally driven disruptions to locally adapted populations (Capblancq et al., 2020), questions of proper application remain (Bay et al., 2018; Fitzpatrick et al., 2018; Hoffmann et al., 2021; Rellstab et al., 2021). Notably, neither its application to demographically representative genetic datasets nor its ability to predict the fitness of a genotype when translocated to a new environment has been thoroughly evaluated using “truth‐known” simulations (i.e., analysis validation, *sensu* Lotterhos et al., 2018).

Gradient Forest differs from genotype–environment association (GEA) analyses (Hoban et al., 2016; Rellstab et al., 2015), which emphasize the identification of environmentally associated alleles, typically using linear univariate approaches (Waldvogel, Schreiber, et al., 2020). GF is a nonparametric multivariate approach that fits an ensemble of regression trees using Random Forest (Breiman, 2001) and then constructs *cumulative importance turnover* functions (see Table 1 for definitions) from these models by determining how well partitions distributed at numerous “split values” along each gradient explain changes in allele frequencies on either side of a split. These cumulative importance curves are generated for each fitted response (e.g., a single‐nucleotide polymorphism (SNP), or a single species) and each environmental predictor, which are weighed and combined to produce an aggregate cumulative importance curve for the genome (or a community of species) along each significant predictor. The steepness of a SNP‐level cumulative importance curve should indicate the rate of change in the allele frequency across the environmental gradient, but this remains untested.

**TABLE 1:** Terminology

Terms	Description
Causal environment	Environmental variable that determines the optimal phenotype
CG Fitness*_m_*_,_ *_n_*	Common Garden (CG) Fitness averaged across all individuals from an *m* source location in an *n* transplant (common garden) location
Cumulative importance	The cumulative sum of “split values” from the fitted GF model
Fitness offset	The difference in fitness experienced by moving an organism from its home environment to a foreign one.
*F*_ST causal_	Genetic differentiation between the source population and transplant (common garden) population at loci with alleles that have causal effects on the phenotype
*F*_ST genome_	Genetic differentiation between the source population and transplant (common garden) population across the genome
GF offset	The Euclidean distance between the cumulative importance output by Gradient Forests at one multivariate environment and another multivariate environment
Local adaptation	The difference between the fitness of populations in sympatry and allopatry
Machine learning algorithm	An algorithm that builds a model based on a subsample of the data in order to make predictions for an expanded dataset
Relative fitness in common garden	The relative probability that an individual with a given genotype would be a parent to offspring in the next generation in the common garden environment, given the causal alleles it possesses and the functions relating alleles to phenotype and phenotype to fitness
Turnover	A change in allele frequency or cumulative importance across an environmental gradient
Climate change vulnerability	The extent to which an organism is susceptible to or unable to cope with climate change and includes the magnitude and rate of exposure to climate change, sensitivity to that exposure, and the ability to cope with climate‐related changes through adaptive capacity. See Foden et al. (2019)

In addition to providing inference regarding the nature of allele frequency change along spatial environmental gradients, GF has been proposed as a method to predict the frequency change in locally beneficial alleles needed to maintain current levels of adaptation following a change in environment (Capblancq et al., 2020; Fitzpatrick & Keller, 2015). In essence, GF’s turnover functions provide a means to transform (i.e., rescale) environmental predictors from their original units (e.g., °C, mm) into common units of cumulative importance. The transformed predictors can then be used to calculate expected genetic differences as the Euclidean distance between populations in time and/or space (Gougherty et al., 2021), a distance referred to as “genetic offset” by Fitzpatrick and Keller (2015) and as “genomic vulnerability” by Bay et al. (2018) and Ruegg et al. (2018). We refer to this distance as “GF Offset” to emphasize its estimation from GF and we suggest the term “genomic vulnerability” should be avoided given that (1) it does not fit established definitions of *climate change vulnerability* (Foden et al., 2019) and (2) it is not clear to what extent GF Offset represents vulnerability, however, defined.

Several questions regarding the use of GF Offset as a metric of maladaptation (e.g., assuming increased GF Offset corresponds to increased *fitness offset*) remain unanswered, including how it is affected by neutral demography. For example, changes in allele frequencies could reflect simple genetic drift rather than adaptive signals (Borrell et al., 2020; Fitzpatrick et al., 2018; Hoban et al., 2016; Rellstab et al., 2015). Smaller populations will tend to exhibit greater signatures of drift than larger populations (Buri, 1956; Helgason et al., 2003; Wright, 1929) and potentially greater allele frequency turnover in regions with smaller population sizes, and less turnover in regions with large population sizes. If these gradients in population structure are aligned with environmental gradients, and neutral loci are not properly eliminated by genome scan procedures prior to offset estimation, then they will be reflected in the fitted cumulative importance curves from GF (i.e., steeper slopes where allele frequency turnover is high, and flatter slopes where turnover is low) and therefore could artificially inflate predicted GF Offsets.

In addition, a complexity inherent to interpretations of GF Offset in the context of forecasting maladaptation is that it is a multivariate distance of allele frequencies from a presumed optimum, meaning that regardless of the direction of change (increase or decrease) in allele frequencies across a gradient, GF Offset will always be positive. The underlying assumption being that a population already occupies its adaptive optimum when sampled, and therefore any subsequent change in allele frequency composition will reduce its fitness. However, because fitness could *decrease* or *increase* in response to environmental change, it is unclear how GF Offset actually relates to fitness, especially when considering such complications as multiple adaptive gradients, nonlinear gradients, or neutral demographic processes.

To evaluate GF Offset as a potential measure of fitness offset, we asked the following questions:

(Q1) *Variation in population size (N)*. What effect do neutral processes, operating on a cline in population size across an environmental gradient, have on GF Offset when only neutral loci are considered? We tested the hypothesis that a decrease in population size would result in an increased GF Offset at neutral loci due to increased genetic drift operating in small populations.
(Q2) *Relationship between GF Offset and fitness offset*. Given equal deme sizes in a metapopulation, how well does GF Offset predict changes in fitness when populations experience an immediate environmental change (i.e., with no associated evolutionary change)? We tested the prediction that GF Offset is inversely related to fitness by conducting in silico common garden experiments under monogenic and polygenic architectures.
(Q3) *GF Offset versus other measures of offset*. Given equal deme sizes in a metapopulation, how does GF Offset perform relative to environmental distance or *F_ST_*? We tested the hypothesis that GF Offset outperforms both environmental distance and genetic distance because GF appropriately weights and scales the environmental gradients to reflect their genetic importance.

We tested the performance of GF Offset and other offset measures in their ability to predict fitness of genotypes when transplanted to common gardens (avoiding the confounding longer‐term dynamics of dispersal and gene flow) across the species range in silico. Using SLiM (Haller & Messer, 2019), we simulated different genome architectures that underlie a phenotype, and different relationships between the phenotypic optimum and a single environmental variable. We then evaluated GF Offset under three scenarios: (1) a neutral model with clinal population size across the environment; (2) a monogenic “population genetic” model with adaptation to a single environment; and (3) a polygenic “quantitative genetic” model with two environmentally adaptive traits, each responding to a different environmental gradient. Overall, we find that GF Offset was strongly correlated with fitness offset, but that multiple factors can confound relationships between offset of fitness.

## 2 MATERIALS & METHODS

### 2.1 Thought experiments

To elucidate what drives the shape of the cumulative importance function, we created five allele frequency patterns across a gradient representing an environmental variable and analyzed them in GF: (1) different sampling schemes of a steep allele frequency cline along one or multiple environmental variables, (2) different slopes of allele frequency clines along an environmental variable, (3) different nonmonotonic relationships between allele frequency and an environmental variable, (4) a comparison of linear and nonmonotonic allele frequency relationships with an environmental variable, and (5) a comparison of a linear allele frequency relationship with an environmental variable and the same relationship with noise added by sampling from a normal distribution with 0 mean and variance of 0.1.

### 2.2 Demography for Q1, Q2, & Q3

We generated simulations using SLiM v3.4. Ten‐thousand individuals (*N*) were split across a metapopulation consisting of 100 demes arranged in a 10×10 connectivity matrix (Figure S1). Each deme (*D*_x,y_) was assigned at least one environmental value that could vary over generational time (*E_j_*_,_ *_t_*, where *j* is a deme and *t* is the generation). When two environmental variables were considered, they are referred to as Environment 1 and Environment 2 (*E1_j_*_,_ *_t_* & *E2_j_*_,_ *_t_*). Symmetric migration was simulated between adjacent demes at a per‐generation migration rate (*m*), and each deme contained equal proportions of males and females, with bi‐parental mating producing the subsequent generation.

Ten genomic linkage groups each containing 50K sites were simulated in each individual, for a total of 500K sites per haploid copy of the diploid genome. The neutral mutation rate (*μ*) was 10^−7^ (a metapopulation‐scaled mutation rate N_T_ * μ of 0.001), and a base recombination rate (*r*) of 10^−5^ (N_T_ * r = 0.1) was used to approximate a distance of 50 cM per linkage group. The population‐scaled mutation and recombination rates were chosen to approximate the resolution that would be observed from sampling SNPs from a larger genome, that is, allowing SNPs 50K bases away to be unlinked, while allowing for signatures of linkage to arise within each linkage group (Lotterhos, 2019). All simulation parameters are listed in Table 2.

**TABLE 2:** Model parameters

Parameter	Value	Description
N	10,000	Total population size of the metapopulation
n	100	Individual deme size
m	0.05/0.2	Per‐generation migration rate (single locus/multilocus)
*μ*	1 × 10^−7^	Neutral mutation rate
r	1 × 10^−5^	Recombination rate
d	0.45	Fitness slope parameter (single‐locus model)
*μ*_QTN_	2.5 × 10^−6^	QTN mutation rate (multilocus models)
*σ*_QTN_	0.1	Variation in QTN effect sizes (multilocus models)
*σ*_S_	4.0	Strength of stabilizing selection for first 1000 generations (multilocus models)
*σ*_K_	1.25/4.0	Strength of stabilizing selection after 2000 generations (multilocus cases 1,2,3/multilocus case 4 for second trait)

Simulations were output as tree sequence files (Haller et al., 2019; Haller & Messer, 2019) following a burn‐in period and a period of stable environmental values (the length of these periods is explained below). The software packages msprime and pyslim were used to prepend a simulation of neutrally coalesced ancestry onto the SLiM‐generated file to guarantee every site was fully coalesced (referred to as “recapitation”), and neutral variants were then overlaid on that recapitated file (Haller et al., 2019). We used vcftools to filter for minor allele frequencies above 0.01, which is on the lower range of MAF filtering criteria in adaptation genomics studies (Byrne et al., 2013; Danecek et al., 2011; Linck & Battey, 2019) but ensures that in our multilocus simulations we included more causal loci in our analyses. Of the 10,000 individuals simulated, 10 individuals from each deme (for a total of 1000 individuals) were randomly selected for downstream analysis.

### 2.3 Q1: Variation in population size (N)

**FIGURE 1:**
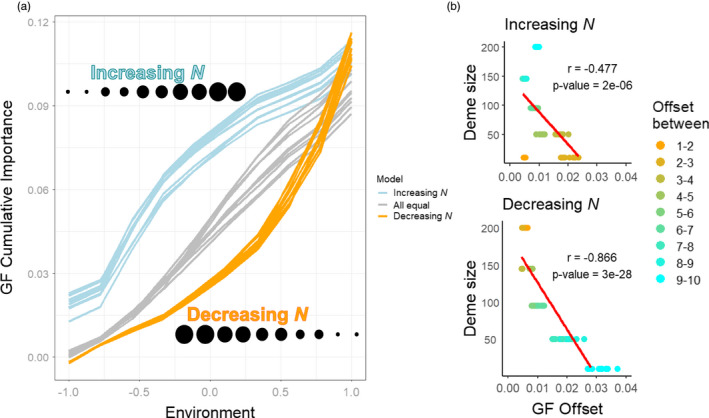
Results of neutral simulations, where no selective pressure is imposed by the underlying environmental clines. (a) The relationship between GF cumulative importance and environment across increasing, decreasing, and equal deme sizes across the environmental gradient. GF Offset increased comparatively more when deme sizes were small. (b). A strong negative linear Pearson's correlation between the GF Offset and deme size in 10 replicates, regardless of the direction of the population gradient. Numbers 1 through 10 in the legend represent the columns in the metapopulation matrix (see Figure S1)

To test the hypothesis that GF Offset at neutral loci could be influenced by variation in genetic drift, we simulated a linear environmental gradient with values from −1 to 1, in three neutral scenarios: equal deme size (*N_d_*); increasing *N_d_*; and decreasing N_d_ (Figure 1). In the equal *N_d_* scenario, each deme contained 100 individuals. In order to maintain a consistent *N_T_* for the increasing and decreasing deme size scenarios, the sum of *N_d_* within each row of the metapopulation grid was made to equal 1000 individuals (e.g., increasing *N_d_* scenario D_1y‐10y_: 10, 10, 50, 50, 95, 95, 145, 145, 200, and 200; the decreasing *N_d_* scenario was the opposite). Each scenario was replicated 10 times.

GF analysis, implemented in R (R Core Team, 2021) in the “gradientForest” package (Ellis et al., 2012), was performed using filtered (MAF > 0.01) allele frequencies and environmental values sampled as described above. We measured the GF Offset of each deme based on an adjacent environmental shift. We tested the null hypothesis of no relationship between *N_d_* and the GF Offset with Pearson's correlation coefficient in R. If GF Offset is not affected by genetic drift, then this correlation will equal 0. However, if higher drift at one end of an environmental cline results in more allele frequency turnover (and higher GF offsets for those populations), then the correlation between *N_d_* and GF Offset will be negative.

### 2.4 Q2 and Q3: Evaluation of offset measures in a locally adapted population

Our goal was to use this next set of simulations to assess the predictive potential of GF Offset for a metapopulation under spatially heterogeneous selection with a straightforward demography (equal deme population size and stepping‐stone migration). These simulations evolved a simple two‐dimensional isolation‐by‐distance population structure. We evaluated the relationship between GF Offset and mean deme fitness, after individuals from that deme were transplanted into another environment in the simulation, for both monogenic and polygenic scenarios. We evaluated offset measures under two genetic architectures: a single‐locus population genetic model, and a multilocus two‐trait quantitative genetic model. In both models, a migration rate of *m* = 0.2 was chosen, which allowed potentially adapted variants to quickly “encounter” their niche and allowed local adaptation to quickly arise across the metapopulation.

We measured the mean local adaptation in each simulation as the difference between mean sympatric fitness (*ω*_S_) and mean allopatric fitness (*ω*_A_) (Blanquart et al., 2013). Mean sympatric fitness was quantified as the average value along the diagonal of the common garden fitness matrix, while mean allopatric fitness was quantified as the average of all of the off‐diagonal values. In order to reduce computation load, we explored best‐case scenarios for GF (e.g., where there is strong environmentally driven local adaptation) that gave high degree of local adaptation, such that the average deme fitness in sympatry was approximately 15%–25% greater than in allopatry (Kawecki & Ebert, 2004).

#### 2.4.1 Single‐locus single‐environment population genetic simulations

In our “population genetic” model of environmental adaptation, a single allele had a linear relationship with fitness across an environmental cline. To avoid a scenario where maladapted individuals persist at the range edge, we modeled individual fitness for each genotype as a function of the environment, with the ancestral allele (*a*) considered to be antagonistically pleiotropic (*sensu* Savolainen et al., 2013) to the emerging derived allele (*A*). See Appendix S1 for more details.

#### 2.4.2 Multilocus two‐trait two‐environment quantitative genetic model

In our “quantitative genetic” two‐trait, two‐environment model of environmental adaptation, QTNs were allowed to arise at a rate of *μ_QTN_* = *μ*/*4* = 2.5 × 10^−6^ (Table 2) across 9 of the 10 linkage groups in the genome, with the final linkage group maintained as a neutral genomic reference. We assumed a quantitative genetic model where alleles contributed additively to two distinct phenotypes for each individual *i* in deme *j* (*P*_1,_ *_ij_* and *P*_2,_ *_ij_*) with no dominance. When an ancestral allele mutated, the bivariate effect size of the derived allele was drawn from a multivariate normal distribution with a standard deviation of *σ*_QTN_ = 0.1 (without covariance) for both traits, which gives flexibility for mutations to evolve with effects on one or both traits (i.e., pleiotropy). For each deme, the phenotypic optimum simply equaled the environmental value.

The relative fitness of individual *i* in deme *j* (*ω_ij_*) was based on how far their *P*_1,_ *_ij_* and *P*_2,_ *_ij_* phenotypes fell from the bivariate optimum in that patch (Θ_1jt_ and Θ_2_ *_jt_*) using a multivariate normal distribution with standard deviation *σ_k_* to represent the strength of stabilizing selection in each deme:

$$\omega_{\mathrm{ij}}=e^{\frac{‐1}{2}\left({\left(\frac{P_{1,ij}‐\Theta_{1jt}}{\sigma_{k}}\right)}^{2}+{\left(\frac{P_{2,ij}‐\Theta_{2tj}}{\sigma_{k}}\right)}^{2}\right)}$$

All relative fitness values were normalized by *ω*_max_, where *P_ij_* = Θ*_jt_*. We ran a cumulative burn‐in period of 3000 generations, consisting of a 1000 generation homogeneous initial burn‐in, followed by a 1000 generation transition burn‐in (see Appendix S1).

**FIGURE 2:**
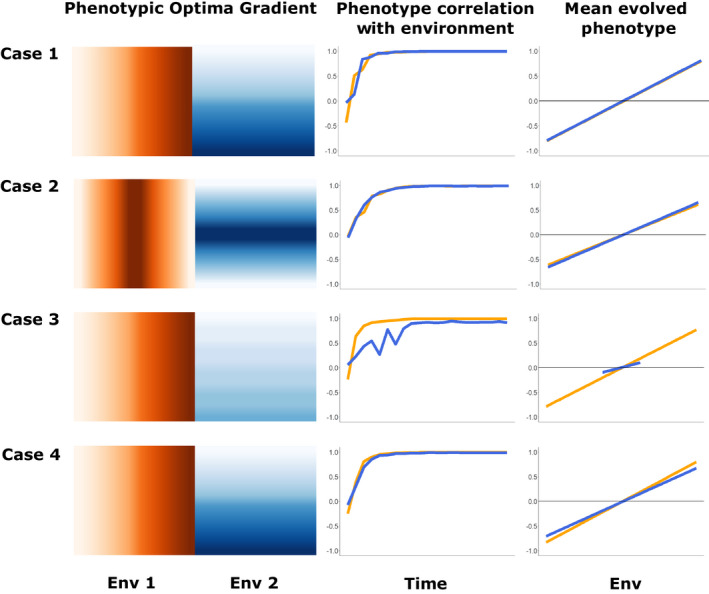
Visualization of the four multilocus case studies. Rows summarize the four key features of each case: the clines of the two phenotypic optima shown as color gradients, with the strength of stabilizing selection listed below each gradient (left column). Strong stabilizing selection (*σ*_K_ value of 1.25) was used for all environments in all cases, except for Case 4, Environment 2, where stabilizing selection was weak (*σ*_K_ value of 4.0). The correlation of each evolved phenotype to its respective environment over time is shown in the middle column; and the mean value of each evolved phenotype at the end of the simulation plotted against its local environment is shown in the far right column. Case 1 demonstrates a “simple” two equal linear environmental gradient scenario, with equally strong selection for each environment. Case 2 has two equal nonlinear environmental gradients, with equally strong selection. Case 3 has two unequal linear gradients, with equal selection. Case 4 has two equal linear gradients, with unequal selection between the two environments

Four cases were considered within the multilocus model (Figure 2). Except as noted, the post–burn‐in optima ranged from −1 to 1 for both environments, and the strength of selection equaled 1.25. Case 1 simulated simple linear clines, in which genetic distance is linearly related to environmental distance. Two orthogonal environmental clines were simulated: environment 1 varying left to right, and environment 2 varying bottom to top (Figure 2 Case 1). Case 2 simulated a situation in which geographic distance does not relate linearly to environmental distance. Here, we simulated two nonmonotonic environments, with the two orthogonal environments increasing from one edge to the middle of the metapopulation, and then decreasing again to the opposite edge. The four corners of the landscape thus had the same environment, but were geographically distant (Figure 2 Case 2). In Case 3, we sought to understand the effect of the slope of the environmental gradient on GF Offset. Case 3 was set up identically to Case 1 except that the optima for environment 2 were narrowed, ranging from −0.25 to 0.25. This was done to produce a narrower phenotypic range in one environment (which should evolve weaker clines in allele frequency), versus another, without changing the strength of selection (Figure 2 Case 3). In Case 4, we sought to determine whether the strength of stabilizing selection affects GF Offset. Case 4 was set up identically to Case 1 except that environment 2 had weaker stabilizing selection, *σ*_k_ = 4 for ϴ_2_ to produce less overall local adaptation, and more additive genetic variation, for trait 2 than produced by Case 1 (Figure 2 Case 4). Each case was replicated 10 times.

### 2.5 Evaluation of offset metrics

In order to represent the relative fitness effects of an instantaneous environmental change, we implemented a reciprocal transplant fitness assessment in our single‐ and multilocus simulations. The mean relative fitness of individuals from a home location *i* (*D*_x,y_) in a transplant “common garden” location *j* (*D*_x’,y’_) was calculated for each deme across all contemporary environments in the simulation, resulting in a 100 × 100 matrix of pairwise relative fitness comparisons. For each common garden *j*, we could then assess how well an offset measure between *i* and *j* predicted the relative fitness of individuals from *i* when transplanted to *j*. We refer to this relative fitness measure as *CG Fitness* (common garden fitness).

For each common garden, the performance of each offset measure was evaluated as the correlation between the offset measure and CG fitness, with a higher correlation inferring that the given offset measure was better able to predict the relative fitness of a genotype when transplanted to the common garden environment. To capture limitations affecting most empirical studies, we used a subset of demes in the reciprocal transplant that was used to calculate the correlation for each common garden (32 demes, Figure S1). From this, it became apparent that the relationship between GF Offset and CG Fitness could depend on the common garden location, so we present the average evaluation performance for common gardens located in the range edge (“Edge”) and range core (“Core”) separately.

#### 2.5.1 Offset metrics

In order to assess the performance of GF Offset in predicting CG Fitness relative to other distance metrics, we also calculated offset measures based on pairwise (i) *F*_ST_ or (ii) environmental distance.

**(i) F_ST_ offset**. F_ST_ offset for a set of individuals from a deme was based on the pairwise *F*_ST_ between the source deme and transplant deme. Pairwise Weir–Cockerham *F*_ST_ values were calculated for every combination of demes at the end of each simulation after filtering loci for MAF > 0.01: one set of such values using all loci (*F*_ST Genome_), another set using only the QTN loci (*F*_ST Causal_).

**(ii) Environmental offset**. Environmental offset for a set of individuals from a deme was based on the pairwise environmental distance between the source location and the transplant location. Environmental distances were calculated using both *n*‐dimensional Euclidean (Cauchy, 1882) and Mahalanobis (*M_D_*, Mahalanobis, 1930) distances between all pairwise demes. We also evaluated the effect of adding noncausal environmental variables into the offset calculations by simulating an additional 12 environmental gradients. Two of these gradients were correlated with the two causal gradients, derived by adding random draws from a univariate normal (μ = 0, σ = 1.3, to allow for a Pearson's correlation between 0.4 and 0.5). The other 10 gradients were drawn from a multivariate normal distribution using a covariance matrix generated by sampling the correlation among variables from a uniform distribution (clusterGeneration package v1.3.4), which gave them a correlation structure similar to that observed in climate data. Distances were calculated using all environmental variables (“total environmental distance,” *M_D_*_‐_ *_all_*, *E_D_*_‐_ *_all_*, 14 variables), and using just the causal environments (“causal environmental distance,” *M_D_*_‐_ *_causal_*, *E_D_*_‐_ *_causal_*, two variables).

**(iii) GF offset**. GF outputs an individual cumulative importance curve for each locus considered to have an environmental association and a weighted aggregate function across all such loci, for each important environmental variable. GF Offset is defined as the Euclidean distance between two locations *A* and *B* in the rescaled environmental space obtained by applying the fitted GF model to the environmental predictors. Note that the rescaling and calculation of GF Offset can be performed using the individual cumulative importance curves (if the goal is to calculate the offset for a single locus, e.g., Keller et al. (2018)), but most applications to date have used the aggregate cumulative importance curves and therefore calculated a multilocus offset:

$$GF\;Offset=\sqrt{\sum {\left(CI_{\mathrm{Ai}}‐CI_{\mathrm{Bi}}\right)}^{2}}$$

where *CI_Ai_* is the multivariate cumulative importance calculated at point *A* for environment *j*, and *CI_Bi_* is the same variable calculated at point *B*. To evaluate the influence of different sets of SNPs or environments used for the calculation, after filtering (MAF > 0.01) we calculated GF Offset based on (i) all loci and all environments (“GF Offset genome, all env.”), (ii) all loci and causal environments (“GF Offset genome, causal env.”), (iii) QTN loci and all environments (“GF Offset causal, all env.”), and (iv) QTN loci and causal environments (“GF Offset causal, causal env.”).

## 3 RESULTS

### 3.1 Thought experiments

**FIGURE 3:**
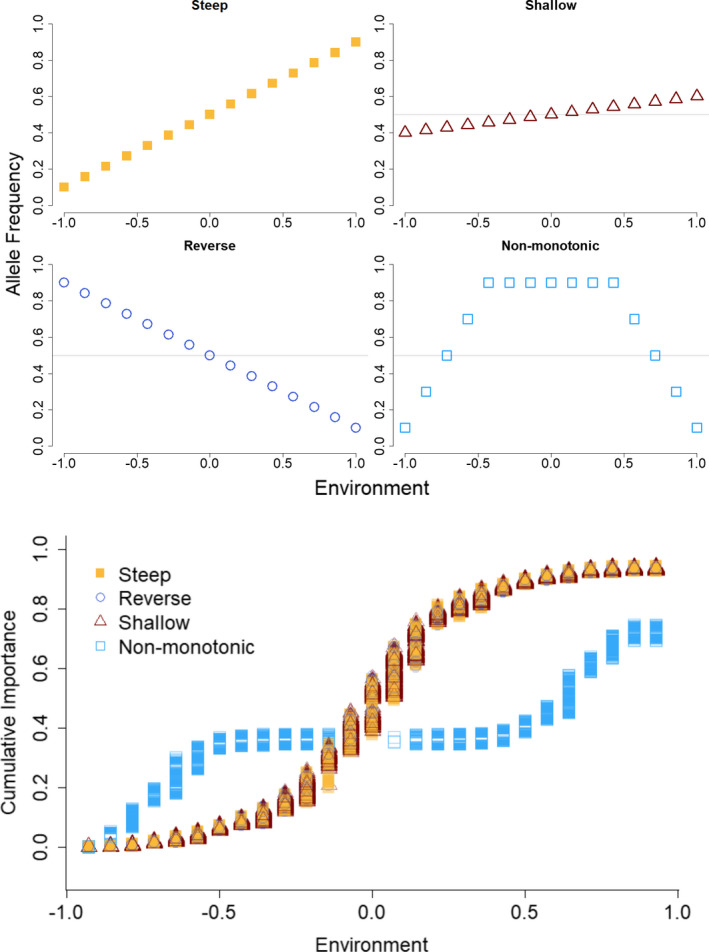
Different allele frequency gradients (steep, shallow, reversed, and nonmonotonic) with respect to an environmental gradient, and their corresponding cumulative importance curve produced by Gradient Forest. Steep, shallow, and reversed gradient cumulative importance values all showed a similar sigmoidal relationship to environment, while the nonmonotonic cumulative importance showed a distinct relationship to environment, increasing only at the edges and remaining flat through the central environmental gradient

To explore the behavior of GF, we created different relationships between genetic variation and an environmental cline and used these to evaluate the relationships between the rate of allele frequency turnover and total amount of cumulative importance, as well as the shape of the cumulative importance curve. In our study, GF produced similar nonlinear cumulative importance curves for linear clines, in which the rate of turnover was highest near the middle of the cline and low elsewhere, regardless of the slope between allele frequency and the environment (Figure 3, “steep,” “reverse,” and “shallow” clines). Nonmonotonic allele frequency patterns also produced a nonlinear cumulative importance curve, the shape of which matched the rate of turnover in allele frequencies, namely for values of the environment where there is rapid turnover in allele frequencies, the slope of the curve is high, and in contrast, where there is no allele frequency turnover in the nonmonotonic case, there is also no increase in the cumulative importance (Figure 3, “non‐monotonic”). Note how in the “non‐monotonic case” the allele frequencies are the same at the environmental extremes (−1 and 1), but a deme would be predicted to have a nonzero GF Offset for an environmental shift from −1 to 1 for that locus. See Appendix S2 for results from a more comprehensive set of thought experiments, which illustrate how the number of sampled populations and random error influence cumulative importance curves (and thus GF Offset).

### 3.2 Q1: Variation in population size (N)

When deme sizes across the environment were uniform, the cumulative importance increased linearly in all replicates (Figure 1a). In contrast, when deme sizes increased or decreased along the environmental gradient, the rate of increase in the cumulative importance was steeper at smaller deme sizes and less steep at larger deme sizes (Figure 1). In all replicates, regardless of whether deme sizes increased or decreased along the environmental gradient, GF Offset was negatively correlated with deme size (Figure 1b).

### 3.3 Q2: Relationship between GF Offset and fitness offset

#### 3.3.1 Single‐locus case

When a single locus of large effect drives environmental adaptation, GF readily identified the environmental gradient driving the clinal pattern (Figure S2). The correlation between GF Offset and CG Fitness was negatively correlated in common gardens at the edges of the range. While the range center also showed a significant negative correlation, the variation in relative fitness was much reduced compared to the edge demes, despite a similar range of offset values (Figure S3).

#### 3.3.2 Multilocus cases

All simulated cases produced high degrees of local adaptation (Figure S4), with Case 3 having the lowest (0.169 – on average relative fitness in sympatry was 16.9% higher than in allopatry) and Case 1 having the highest (0.288). Across all cases, both traits evolved high correlations between phenotype and environment (Figure 2). On average, approximately 1600 QTN loci evolved in these simulations, indicating that the genetic architecture of the traits was highly polygenic. After MAF filtering, a little over 100 loci remained, but those retained loci typically explained 50%–60% of the total additive genetic variance for each trait (additive genetic variance for locus *i* on a trait *j* was approximated as α*_ij_* 2*p_i_* (1‐*p_i_*), where *α_ij_* was the effect of locus *i* on trait *j*, and *p_i_* was the frequency of locus *i* in the metapopulation postsampling), indicating that a large number of QTNs were rare alleles that individually contributed little to the total additive genetic variance.

We evaluated if GF could identify the causal environments when all the simulated environments were input. In the cases with two linear causal environmental clines (Cases 1, 3, & 4), GF was able to identify the causal environment driving adaptation (Figure S5). However, when two nonlinear causal environments were simulated (Case 2), they were not ranked as most important when all alleles were considered (see Figure S5).

**FIGURE 4:**
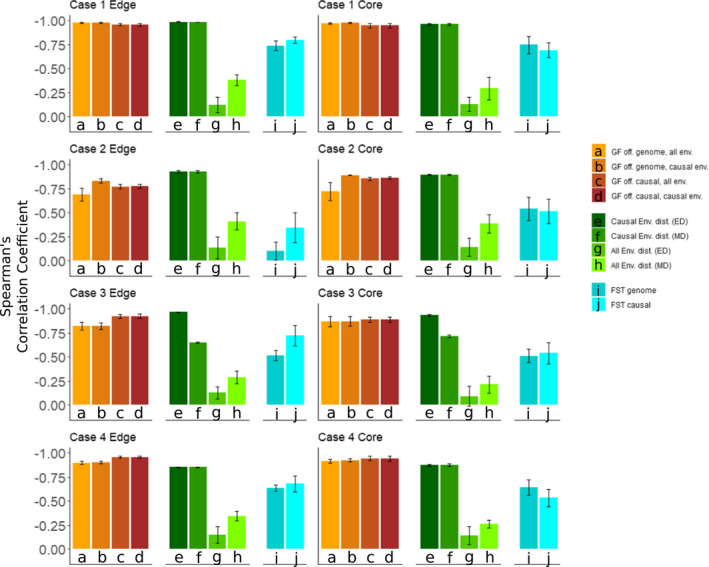
Spearman's correlation between Common Garden (CG) Fitness and different measures of offset (GF Offset = browns, Environmental distances = greens, *F*_ST_ = blues), for common gardens at the edge of the landscape (left column) and common gardens in the center (core) of the landscape (right column). Euclidean environmental distance = ED, Mahalanobis environmental distance = MD. Each row represents an individual case of the multilocus simulation: first row shows Case 1, where both causal environments were linear orthogonals; second row Case 2, where both causal environments were nonlinear orthogonal “mountain peaks”; third row Case 3, where the optimal phenotypic range for trait 2 was narrower than for trait 1; and fourth row Case 4, where the strength of selection on trait 2 was weaker than on trait 1

Gradient Forest Offset had a consistent negative correlation with CG Fitness across all replicates for all four multilocus cases, in both Core and Edge demes, with similar performance regardless of whether all loci or only causal loci were used (orange bars, Figure 4). While GF Offset did not perform as well as the causal environmental distance (dark green bars, Figure 4), it consistently outperformed overall environmental distance (light green bars, Figure 4), *F*_ST Genome_, and *F*_ST Causal_ (light blue bars, Figure 4) as a predictor of CG Fitness.

### 3.4 Q3: GF Offset versus other measures of offset

#### 3.4.1 Environmental distance as a predictor of relative fitness across multilocus cases

Between the two environmental distance measures, *M_D_* performed just as well as *E_D_* in all cases except Case 3, where the internal standardization of each environment by its variance caused the second environmental variable with lower variance to bias *M_D_*.

#### 3.4.2 Genetic distance as a predictor of relative fitness across multilocus cases

Measures of *F*_ST Genome_ and *F*_ST Causal_ generally were among the poorest predictors of CG Fitness across all cases, but did perform slightly better than overall environmental distance. Offset predictions by both measures of *F*_ST_ were more impacted by deme location (core vs. edge) than any other method. While *F*_ST Causal_ was generally a better predictor of CG Fitness overall for the Edge demes, *F*_ST Genome_ performed similarly in the Core demes.

## 4 DISCUSSION

Potential applications for GF Offset range from small‐scale conservation efforts in genetic rescue (Houde et al., 2015), assisted migration for agricultural and forestry for increased production (Aitken & Bemmels, 2016), and predicting and/or forecasting the potential for maladaptation under climate change (Capblancq et al., 2020). The work present here provides some initial simulation testing concerning the application of GF Offset.

Whether the intent is to predict how a population will perform when moved to another location or when faced with environmental changes in its current location, GF Offset is useful only to the extent that it approximates associated reductions in fitness (or maladaptation). Across all simulated cases with selection, GF Offset performed well at predicting CG Fitness, regardless of whether or not nonadapted loci and noncausal environments were included in the analysis. However, it is important to note that we used GF offset to predict relative fitness in our simulations, as absolute fitness was not simulated. GF Offset was representative of changes in *relative* fitness under both the simulated single‐locus and polygenic architectures, lending support to the key assumption of a negative relationship between GF Offset and fitness that underlies the use of GF for predicting maladaptation. When all environmental variables were considered (causal and noncausal), GF Offset, which is based on weighting of environmental gradients given the strength of their association with adaptive variation, outperformed predictions of changes in relative fitness from the unweighted distance metrics. However, when environmental drivers of adaptation were known and only those gradients were included in the offset calculation, environmental distance performed as well as, or better, than GF Offset. Note that the demography in those simulations was simple in the sense of equal deme sizes and migration rates. We also found that neutral demography can confound GF Offset, and that GF Offset can be sensitive to sampling schemes. Additional research is needed to inform how to account for population structure and apply appropriate filtration thresholds prior to calculations of offset measures, especially if they are to be applied to real‐world applications.

### 4.1 Interpretation and comparisons of CG Fitness correlations

The strength of the relationship between causal environmental distance and CG Fitness underscores the need to identify environmental drivers of local adaptation when attempting to predict fitness under changing environments. While one study has found a positive relationship between the strength of genotype–environment associations and environments that predict common garden fitness (Mahony et al., 2020), a strength of GF is its ability to identify linear selective environments from multiple candidate environmental variables. However, GF did not rank selective environments as most important in all cases; see Figure S5. GF Offset consistently was a better predictor of CG Fitness than *F*_ST_, regardless of whether *F*_ST_ was calculated using only adapted loci or not. This has significant implications for the use of *F*_ST_ as a decision metric for prioritizing conservation efforts, which other evaluations have also shown has limitations (Xuereb et al., 2020). In addition, we observed that offset metrics based on whole‐genome data performed similarly to data filtered for only the known causal alleles, for both GF offset and *F_ST_*. A large number of rare causal alleles were removed from the dataset by the 0.01 MAF filtration threshold, which might explain why offset metrics based on known causal alleles were not a better predictor of relative fitness offset than those based on whole‐genome data, where linked loci could have contributed a stronger overall signal. It should also be noted that the magnitude of GF Offset cannot be compared across different studies, as there is no currently accepted approach to standardize the measure (e.g., to account for differences in the number of variables used in the analysis, see range of offset values in supplemental results in Appendix S1).

### 4.2 Conceptual concerns with GF offset

Although GF Offset is increasingly used to predict maladaptation, we do not fully understand its performance in natural systems. The underlying assumptions in predictive applications of GF Offset, as with most other approaches to fitness inference, are that a population already occupies its adaptive optimum when sampled, and therefore changes in allele frequency composition (regardless of direction) result in decreases in fitness. Furthermore, GF Offset assumes that the molecular signatures of local adaptation when multiple demes occupy the same environment at different sites are the same. These assumptions are likely met when there is high stability of both the adaptive landscape and the genomic architecture maintaining fitness. On the other hand, these assumptions are likely to be violated with shifts in the adaptive landscape potentially driven by fluctuations in climate and ecology (Arnold et al., 2001), and under transient, highly redundant genomic architectures (Láruson et al., 2020). With the single locus of large effect simulation showing the strongest relationship between GF Offset and CG Fitness (Figure S3), and the nonlinear environment simulation (Case 2) the weakest, GF Offset is clearly impacted by both genomic architecture and the environmental landscape of the metapopulation. Also, for linear allele frequency clines, the steepness of the cline did not influence the (nonlinear) cumulative importance curve. Although when the cline was nonmonotonic, the cumulative importance curve better matched the pattern of turnover. Therefore, at least for linear clines, the interpretation of the steepness of the cumulative importance curve as a measure of the rate of allele frequency change may not be appropriate. Rather, the cumulative importance curves reflect how much of the variation in allele frequencies is explained by the environmental gradient, regardless of the absolute difference in allele frequencies along the gradient.

### 4.3 Caveats and best practices

The sensitivity of GF Offset to deme size requires special consideration when studying populations that do not maintain a uniform distribution across their range, especially for populations of conservation concern (Borrell et al., 2020; Rellstab et al., 2015). Empirical studies have found negative associations between GF Offset and population size (Bay et al., 2018; Ruegg et al., 2018), but our results show that these associations can arise due to neutral genetic drift and not signals of selection as assumed. At small (large) deme sizes, there is more (less) genetic drift, which leads to greater (less) allele frequency turnover at that end of the environmental gradient, and therefore more (less) rapid increases in cumulative importance and larger (smaller) GF Offset. Thus, empirical studies that find a correlation between population size and offset values should not be considered examples of validation for offset measures. Additionally, datasets where deme size and environments (with high importance in GF) are correlated would be most susceptible to this phenomenon, and investigators should report these relationships.

These results highlight that empirical observed negative relationships between GF Offset and population size cannot be assumed to indicate a selection‐driven response, and underscores the need to account for population structure during genome scans for selection prior to fitting GF. Because our results illustrate that genetic drift can confound measures of GF Offset, it is likely that more complex demographic processes, such as population size fluctuations, variable gene flow, admixture, or secondary contact, will also confound GF Offset, as they have been shown to confound genome scans (e.g., Harris et al., 2018; Lotterhos & Whitlock, 2014; Luu et al., 2017). Note that in our neutral simulation case, it is not clear how many of these neutral loci would have been eliminated by implementing a genome scan for selection prior to offset calculation, as has been advocated for previously (Capblancq et al., 2020). The effects of increased demographic complexity in conjunction with adaptive processes on GF Offset have not been fully explored here, and is an important direction for future research. Since other metrics of genetic offset have been found to be associated with population size (Borrell et al., 2020), the potential effect of genetic drift on various other offset measures should also be more fully evaluated. To this end, recent studies have explored correcting allele frequencies for population structure based on the population covariance matrix (Berg & Coop, 2014) prior to analysis with GF for outlier detection (Fitzpatrick et al., 2021).

In our simulations, the degree of negative correlation between GF Offset and relative fitness depends on sampling scheme, genetic architecture, the genotype–phenotype–fitness map, and the pattern of environmental variation on the landscape. The thought experiments showed that GF can be sensitive to sampling schemes and has higher performance when populations are densely sampled along environmental gradients. This raises questions about how sampling schemes might bias environmental predictor importance values and requires further study. Sampling considerations are further impacted by the way GF trains itself on approximately two‐thirds of the input number of populations, albeit repeatedly, so GF’s ability to confidently predict the training data can be impacted when only a few populations are analyzed.

### 4.4 Opportunities for future development

A key limitation of our simulation to highlight is that all fitness values were calculated as relative fitness, whereas most conservation minded applications of GF will be concerned with absolute fitness (i.e., population size may be shrinking with increasing genotype–environment mismatches). This distinction between absolute and relative fitness is critical when using models to inform conservation management decisions, since changes in allele frequencies (due to genetic drift or differences in relative fitness) do not necessarily impact demography as absolute fitness does. In fact, allele frequency changes can *only ever* reflect relative fitness (Brady et al., 2019). For example, a novel genotype might be increasing in a deme because it has higher relative fitness than another genotype, but both the demes could still be declining in size because both genotypes have low absolute fitness. Therefore, the rationale that allele frequency changes would be useful in making fitness predictions under climate change needs closer examination, and is an important area for future research.

To assess the predictive potential of GF Offset, our simulations focused on a select few “idealized” scenarios (i.e., high local adaptation, all relevant causal environmental measures included, and fitness assessment across all common gardens at a fixed time point), with corresponding data which are unlikely to be reflected in empirical work. All calculations using causal alleles were not dependent on those alleles being identified as outliers – it was simply assumed that they were known. In reality, even though many causal alleles showed relatively elevated *F*_ST_ values (Figure S6), most would be unlikely to be identified through any outlier cut‐off approach. However, even when we employed the nonideal practice of including all loci, with no attempt at filtering for alleles under selection, GF Offset remained highly correlated with our measure of CG Fitness. Future studies should simulate less ideal scenarios including more complex demographies, non‐Wright–Fisher dynamics (with variable deme sizes), and errors in genotyping or outlier detection.

## 5 CONCLUSIONS

While most emergent complexities involved in applying GF Offset to realistic scenarios are still poorly understood, there is still promise in the application of this method to identifying key environmental drivers of local adaptation and for estimating fitness declines in response to rapid environmental change. This may be especially applicable to translocation assessments of at‐risk species or cultivars, and genetic rescue efforts. Key considerations of demography, genomic architecture, and the nature of environmental gradients have been highlighted here, as in earlier work (Capblancq et al., 2020; Fitzpatrick et al., 2021; Gougherty et al., 2021), as factors that can have significant effects on measures of GF Offset. All future inferences drawn from the potential negative relationship between GF Offset and fitness must take care to address these features of the study system explicitly, and acknowledge the limitations of all inferences if any of these factors are not well understood.

## CONFLICT OF INTERESTS

The authors have no conflict of interests to declare.

## Supporting information

Fig S1

Fig S2

Fig S3

Fig S4

Fig S5

Fig S6

Appendix S1

Appendix S2

## Data Availability

Simulation code, resultant data, and analysis code for reproducing the presented results will be found at the Dryad repository: *Data from Seeing the Forest for the trees*: *Assessing genetic offset predictions from Gradient Forest*. https://doi.org/10.5061/dryad.x95x69pkk.

## References

[eva13354-bib-0001] Aitken, S. N. , & Bemmels, J. B. (2016). Time to get moving: Assisted gene flow of forest trees. Evolutionary Applications, 9, 271–290. 10.1111/eva.12293 27087852 PMC4780373

[eva13354-bib-0002] Aitken, S. N. , Yeaman, S. , Holliday, J. A. , Wang, T. , & Curtis‐McLane, S. (2008). Adaptation, migration or extirpation: Climate change outcomes for tree populations. Evolutionary Applications, 1, 95–111. 10.1111/j.1752-4571.2007.00013.x 25567494 PMC3352395

[eva13354-bib-0003] Arnold, S. J. , Pfrender, M. E. , & Jones, A. G. (2001). The adaptive landscape as a conceptual bridge between micro‐ and macroevolution. Genetica, 112–113, 9–32. 11838790

[eva13354-bib-0004] Bay, R. A. , Harrigan, R. J. , Underwood, V. L. , Gibbs, H. L. , Smith, T. B. , & Ruegg, K. (2018). Genomic signals of selection predict climate‐driven population declines in a migratory bird. Science, 359, 83–86. 10.1126/science.aan4380 29302012

[eva13354-bib-0005] Berg, J. J. , & Coop, G. (2014). A population genetic signal of polygenic adaptation. PLoS Genetics, 10, e1004412. 10.1371/journal.pgen.1004412 25102153 PMC4125079

[eva13354-bib-0006] Bland, L. M. , Collen, B. , Orme, C. D. L. , & Bielby, J. (2015). Predicting the conservation status of data‐deficient species. Conservation Biology, 29, 250–259. 10.1111/cobi.12372 25124400

[eva13354-bib-0007] Blanquart, F. , Kaltz, O. , Nuismer, S. L. , & Gandon, S. (2013). A practical guide to measuring local adaptation. Ecology Letters, 16, 1195–1205. 10.1111/ele.12150 23848550

[eva13354-bib-0008] Borrell, J. S. , Zohren, J. , Nichols, R. A. , & Buggs, R. J. A. (2020). Genomic assessment of local adaptation in dwarf birch to inform assisted gene flow. Evolutionary Applications, 13, 161–175. 10.1111/eva.12883 31892950 PMC6935589

[eva13354-bib-0009] Brady, S. P. , Bolnick, D. I. , Barrett, R. D. H. , Chapman, L. , Crispo, E. , Derry, A. M. , Eckert, C. G. , Fraser, D. J. , Fussmann, G. F. , Gonzalez, A. , Guichard, F. , Lamy, T. , Lane, J. , McAdam, A. G. , Newman, A. E. M. , Paccard, A. , Robertson, B. , Rolshausen, G. , Schulte, P. M. , … Hendry, A. (2019). Understanding maladaptation by uniting ecological and evolutionary perspectives. The American Naturalist, 194, 495–515. 10.1086/705020 31490718

[eva13354-bib-0010] Breiman, L. (2001). Random forests. Machine Learning, 45, 5–32.

[eva13354-bib-0011] Buri, P. (1956). Gene frequency in small populations of mutant *Drosophila* . Evolution, 10, 367–402.

[eva13354-bib-0012] Byrne, S. , Czaban, A. , Studer, B. , Panitz, F. , Bendixen, C. , & Asp, T. (2013). Genome wide allele frequency fingerprints (GWAFFs) of populations via genotyping by sequencing. PLoS One, 8, e57438. 10.1371/journal.pone.0057438 23469194 PMC3587605

[eva13354-bib-0013] Capblancq, T. , Fitzpatrick, M. C. , Bay, R. A. , Exposito‐Alonso, M. , & Keller, S. R. (2020). Genomic prediction of (Mal)Adaptation across current and future climatic landscapes. Annual Review of Ecology Evolution and Systematics, 51, 245–269. 10.1146/annurev-ecolsys-020720-042553

[eva13354-bib-0014] Cauchy, A. L. (1882). Oeuvres complètes d’Augustin Cauchy. Gauthier‐Villars.

[eva13354-bib-0015] Danecek, P. , Auton, A. , Abecasis, G. , Albers, C. A. , Banks, E. , DePristo, M. A. , Handsaker, R. E. , Lunter, G. , Marth, G. T. , Sherry, S. T. , McVean, G. , & Durbin, R. (2011). The variant call format and VCFtools. Bioinformatics, 27, 2156–2158. 10.1093/bioinformatics/btr330 21653522 PMC3137218

[eva13354-bib-0016] Ellis, N. , Smith, S. J. , & Roland Pitcher, C. (2012). Gradient forests: Calculating importance gradients on physical predictors. Ecology, 93(1), 156–168. 10.1890/11-0252.1 22486096

[eva13354-bib-0017] Fitzpatrick, M. C. , Chhatre, V. E. , Soolanayakanahally, R. Y. , & Keller, S. R. (2021). Experimental support for genomic prediction of climate maladaptation using the machine learning approach Gradient Forests. Molecular Ecology Resources, 21(8), 2749–2765. 10.1111/1755-0998.13374 33683822

[eva13354-bib-0018] Fitzpatrick, M. C. , & Keller, S. R. (2015). Ecological genomics meets community‐level modelling of biodiversity: mapping the genomic landscape of current and future environmental adaptation. Ecology Letters, 18, 1–16. 10.1111/ele.12376 25270536

[eva13354-bib-0019] Fitzpatrick, M. C. , Keller, S. R. , & Lotterhos, K. E. (2018). Comment on “Genomic signals of selection predict climate‐driven population declines in a migratory bird”. Science, 361, eaat7279. 10.1126/science.aat7279 30072513

[eva13354-bib-0020] Foden, W. B. , Young, B. E. , Akçakaya, H. R. , Garcia, R. A. , Hoffmann, A. A. , Stein, B. A. , Thomas, C. D. , Wheatley, C. J. , Bickford, D. , Carr, J. A. , Hole, D. G. , Martin, T. G. , Pacifici, M. , Pearce‐Higgins, J. W. , Platts, P. J. , Visconti, P. , Watson, J. E. M. , & Huntley, B. (2019). Climate change vulnerability assessment of species. Wiley Interdisciplinary Reviews‐Climate Change, 10, e551. 10.1002/wcc.551

[eva13354-bib-0021] Freer, J. J. , Partridge, J. C. , Tarling, G. A. , Collins, M. A. , & Genner, M. J. (2018). Predicting ecological responses in a changing ocean: The effects of future climate uncertainty. Marine Biology, 165, 7. 10.1007/s00227-017-3239-1 29170567 PMC5680362

[eva13354-bib-0022] Gougherty, A. V. , Keller, S. R. , & Fitzpatrick, M. C. (2021). Maladaptation, migration and extirpation fuel climate change risk in a forest tree species. Nature Climate Change, 11, 166–171. 10.1038/s41558-020-00968-6

[eva13354-bib-0023] Haller, B. C. , Galloway, J. , Kelleher, J. , Messer, P. W. , & Ralph, P. L. (2019). Tree‐sequence recording in SLiM opens new horizons for forward‐time simulation of whole genomes. Molecular Ecology Resources, 19, 552–566. 10.1111/1755-0998.12968 30565882 PMC6393187

[eva13354-bib-0024] Haller, B. C. , & Messer, P. W. (2019). SLiM 3: Forward genetic simulations beyond the Wright‐Fisher Model. Molecular Biology and Evolution, 36(3), 632–637. 10.1093/molbev/msy228 30517680 PMC6389312

[eva13354-bib-0025] Harris, R. B. , Sackman, A. , & Jensen, J. D. (2018). On the unfounded enthusiasm for soft selective sweeps II: Examining recent evidence from humans, flies, and viruses. PLoS Genetics, 14, e1007859. 10.1371/journal.pgen.1007859 30592709 PMC6336318

[eva13354-bib-0026] Helgason, A. , Nicholson, G. , Stefánsson, K. , & Donnelly, P. (2003). A reassessment of genetic diversity in Icelanders: Strong evidence from multiple loci for relative homogeneity caused by genetic drift. Annals of Human Genetics, 67, 281–297. 10.1046/j.1469-1809.2003.00046.x 12914564

[eva13354-bib-0027] Hoban, S. , Kelley, J. L. , Lotterhos, K. E. , Antolin, M. F. , Bradburd, G. , Lowry, D. B. , Poss, M. L. , Reed, L. K. , Storfer, A. , & Whitlock, M. C. (2016). Finding the genomic basis of local adaptation: Pitfalls, practical solutions, and future directions. American Naturalist, 188, 379–397. 10.1086/688018 PMC5457800 27622873

[eva13354-bib-0028] Hoffmann, A. A. , Weeks, A. R. , & Sgrò, C. M. (2021). Opportunities and challenges in assessing climate change vulnerability through genomics. Cell, 184, 1420–1425. 10.1016/j.cell.2021.02.006 33740448

[eva13354-bib-0029] Houde, A. L. S. , Garner, S. R. , & Neff, B. D. (2015). Restoring species through reintroductions: Strategies for source population selection. Restoration Ecology, 23(6), 746–753. 10.1111/rec.12280

[eva13354-bib-0030] Kawecki, T. J. , & Ebert, D. (2004). Conceptual issues in local adaptation. Ecology Letters, 7, 1225–1241. 10.1111/j.1461-0248.2004.00684.x

[eva13354-bib-0031] Keller, S. R. , Chhatre, V. E. , & Fitzpatrick, M. C. (2018). Influence of range position on locally adaptive gene‐environment associations in populus flowering time genes. Journal of Heredity, 109, 47–58. 10.1093/jhered/esx098 29126208

[eva13354-bib-0032] Láruson, Á. J. , Yeaman, S. , & Lotterhos, K. E. (2020). The importance of genetic redundancy in evolution. Trends in Ecology & Evolution, 35, 809–822. 10.1016/j.tree.2020.04.009 32439075

[eva13354-bib-0033] Layton, K. K. S. , Snelgrove, P. V. R. , Dempson, J. B. , Kess, T. , Lehnert, S. J. , Bentzen, P. , Duffy, S. J. , Messmer, A. M. , Stanley, R. R. E. , DiBacco, C. , Salisbury, S. J. , Ruzzante, D. E. , Nugent, C. M. , Ferguson, M. M. , Leong, J. S. , Koop, B. F. , & Bradbury, I. R. (2021). Genomic evidence of past and future climate‐linked loss in a migratory Arctic fish. Nature Climate Change, 11, 158–165. 10.1038/s41558-020-00959-7

[eva13354-bib-0034] Linck, E. , & Battey, C. J. (2019). Minor allele frequency thresholds strongly affect population structure inference with genomic data sets. Molecular Ecology Resources, 19, 639–647. 10.1111/1755-0998.12995 30659755

[eva13354-bib-0035] Lotterhos, K. E. (2019). The effect of neutral recombination variation on genome scans for selection. G3 Genes|Genomes|Genetics, 9(6), 1851–1867. 10.1534/g3.119.400088 30971391 PMC6553532

[eva13354-bib-0036] Lotterhos, K. E. , Moore, J. H. , & Stapleton, A. E. (2018). Analysis validation has been neglected in the age of reproducibility. PLoS Biology, 16, e3000070. 10.1371/journal.pbio.3000070 30532167 PMC6301703

[eva13354-bib-0037] Lotterhos, K. E. , & Whitlock, M. C. (2014). Evaluation of demographic history and neutral parameterization on the performance of FST outlier tests. Molecular Ecology, 23, 2178–2192. 24655127 10.1111/mec.12725 PMC4228763

[eva13354-bib-0038] Luu, K. , Bazin, E. , & Blum, M. G. B. (2017). pcadapt: An Rpackage to perform genome scans for selection based on principal component analysis. Molecular Ecology Resources, 17(1), 67–77. 10.1111/1755-0998.12592 27601374

[eva13354-bib-0039] Mahalanobis, P. C. (1930). On tests and measures of groups divergence. Part I. Theoretical formulae. Journal of the Asiatic Society of Bengal, 26, 541–588.

[eva13354-bib-0040] Mahony, C. R. , MacLachlan, I. R. , Lind, B. M. , Yoder, J. B. , Wang, T. , & Aitken, S. N. (2020). Evaluating genomic data for management of local adaptation in a changing climate: A lodgepole pine case study. Evolutionary Applications, 13, 116–131. 10.1111/eva.12871 31892947 PMC6935591

[eva13354-bib-0041] Matz, M. V. , Treml, E. A. , & Haller, B. C. (2020). Estimating the potential for coral adaptation to global warming across the Indo‐West Pacific. Global Change Biology, 26, 3473–3481. 10.1111/gcb.15060 32285562

[eva13354-bib-0042] Nunez, S. , Arets, E. , Alkemade, R. , Verwer, C. , & Leemans, R. (2019). Assessing the impacts of climate change on biodiversity: is below 2°C enough? Climatic Change, 154, 351–365.

[eva13354-bib-0043] Pacifici, M. , Foden, W. B. , Visconti, P. , Watson, J. E. M. , Butchart, S. H. M. , Kovacs, K. M. , Scheffers, B. R. , Hole, D. G. , Martin, T. G. , Akçakaya, H. R. , Corlett, R. T. , Huntley, B. , Bickford, D. , Carr, J. A. , Hoffmann, A. A. , Midgley, G. F. , Pearce‐Kelly, P. , Pearson, R. G. , Williams, S. E. , … Rondinini, C. (2015). Assessing species vulnerability to climate change. Nature Climate Change, 5, 215–224. 10.1038/nclimate2448

[eva13354-bib-0044] R Core Team . (2021). R: A language and environment for statistical computing. R Foundation for Statistical Computing. https://www.R‐project.org/

[eva13354-bib-0045] Razgour, O. , Taggart, J. B. , Manel, S. , Juste, J. , Ibáñez, C. , Rebelo, H. , Alberdi, A. , Jones, G. , & Park, K. (2018). An integrated framework to identify wildlife populations under threat from climate change. Molecular Ecology Resources, 18, 18–31. 10.1111/1755-0998.12694 28649779 PMC6849758

[eva13354-bib-0046] Rellstab, C. , Dauphin, B. , & Exposito‐Alonso, M. (2021). Prospects and limitations of genomic offset in conservation management. Evolutionary Applications, 14, 1202–1212. 10.1111/eva.13205 34025760 PMC8127717

[eva13354-bib-0047] Rellstab, C. , Gugerli, F. , Eckert, A. J. , Hancock, A. M. , & Holderegger, R. (2015). A practical guide to environmental association analysis in landscape genomics. Molecular Ecology, 24, 4348–4370. 10.1111/mec.13322 26184487

[eva13354-bib-0048] Ruegg, K. , Bay, R. A. , Anderson, E. C. , Saracco, J. F. , Harrigan, R. J. , Whitfield, M. , Paxton, E. H. , & Smith, T. B. (2018). Ecological genomics predicts climate vulnerability in an endangered southwestern songbird. Ecology Letters, 21, 1085–1096. 10.1111/ele.12977 29745027

[eva13354-bib-0049] Savolainen, O. , Lascoux, M. , & Merilä, J. (2013). Ecological genomics of local adaptation. Nature Reviews Genetics, 14, 807–820. 10.1038/nrg3522 24136507

[eva13354-bib-0050] Urban, M. C. , Bocedi, G. , Hendry, A. P. , Mihoub, J.‐B. , Pe’er, G. , Singer, A. , Bridle, J. R. , Crozier, L. G. , De Meester, L. , Godsoe, W. , Gonzalez, A. , Hellmann, J. J. , Holt, R. D. , Huth, A. , Johst, K. , Krug, C. B. , Leadley, P. W. , Palmer, S. C. F. , Pantel, J. H. , … Travis, J. M. J. (2016). Improving the forecast for biodiversity under climate change. Science, 353, 1113. 10.1126/science.aad8466 27609898

[eva13354-bib-0051] Waldvogel, A.‐M. , Feldmeyer, B. , Rolshausen, G. , Exposito‐Alonso, M. , Rellstab, C. , Kofler, R. , Mock, T. , Schmid, K. , Schmitt, I. , Bataillon, T. , Savolainen, O. , Bergland, A. , Flatt, T. , Guillaume, F. , & Pfenninger, M. (2020). Evolutionary genomics can improve prediction of species’ responses to climate change. Evolution Letters, 4(1), 4–18. 10.1002/evl3.154 32055407 PMC7006467

[eva13354-bib-0052] Waldvogel, A.‐M. , Schreiber, D. , Pfenninger, M. , & Feldmeyer, B. (2020). Climate change genomics calls for standardized data reporting. Frontiers in Ecology and Evolution, 8, 242. 10.3389/fevo.2020.00242.

[eva13354-bib-0053] Wright, S. (1929). The evolution of dominance. The American Naturalist, 63(689), 556–561. 10.1086/280290 29585648

[eva13354-bib-0054] Xuereb, A. , D’Aloia, C. C. , Andrello, M. , Bernatchez, L. , & Fortin, M.‐J. (2020). Incorporating putatively neutral and adaptive genomic data into marine conservation planning. Conservation Biology, 35(3), 909–920. 10.1111/cobi.13609 32785955

