# Supplementary figures and images for: Seeing the forest for the trees: Assessing genetic offset predictions from gradient forest
Source: Evol Appl. 2022 Feb 25;15(3):403–16. doi: 10.1111/eva.13354 (PMC8965365; doi:10.1111/eva.13354)

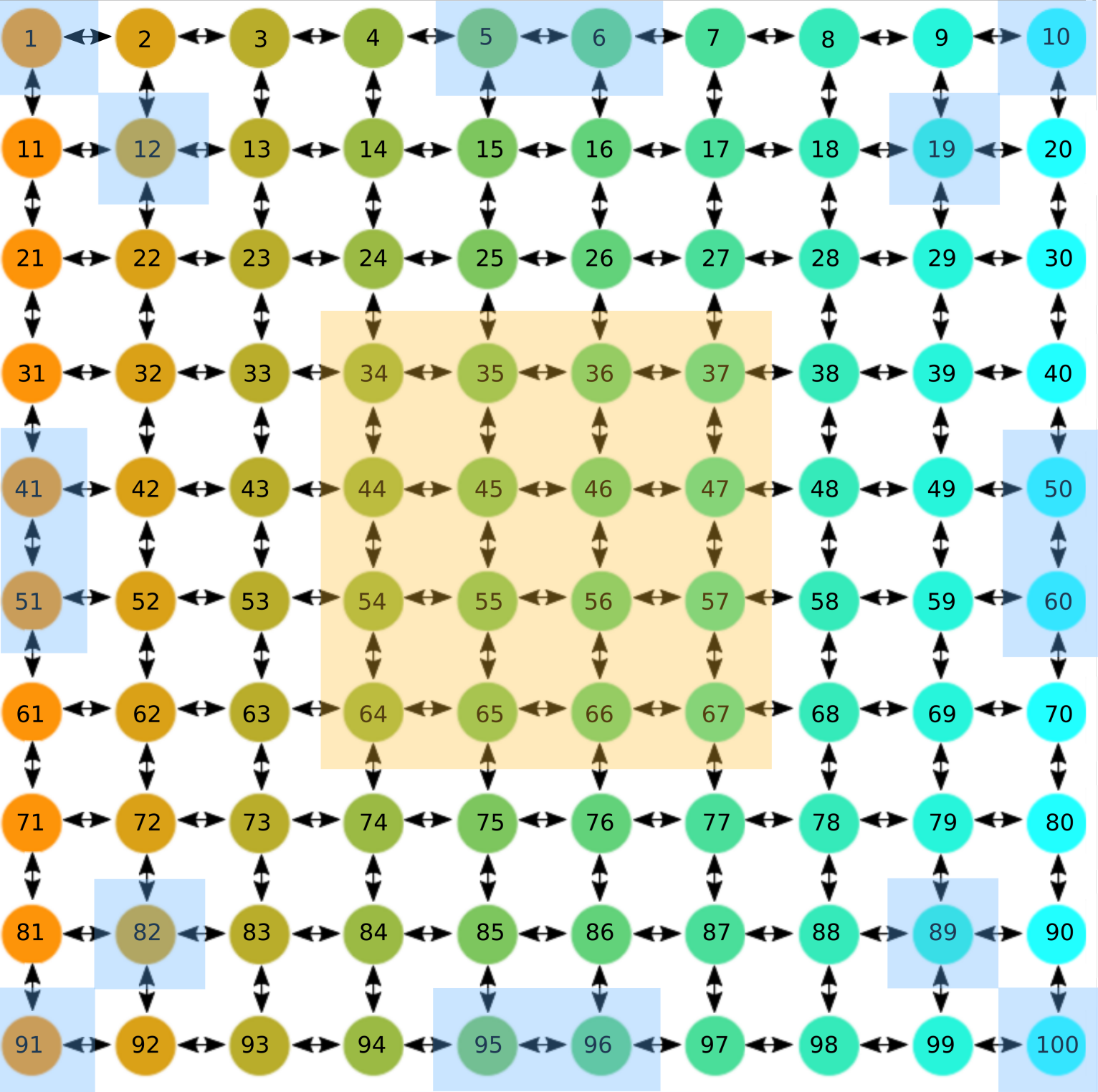

Supplement: Supplementary file 1 — Fig S1 [file EVA-15-403-s002.pdf]

A

 $R^2$  weighted importance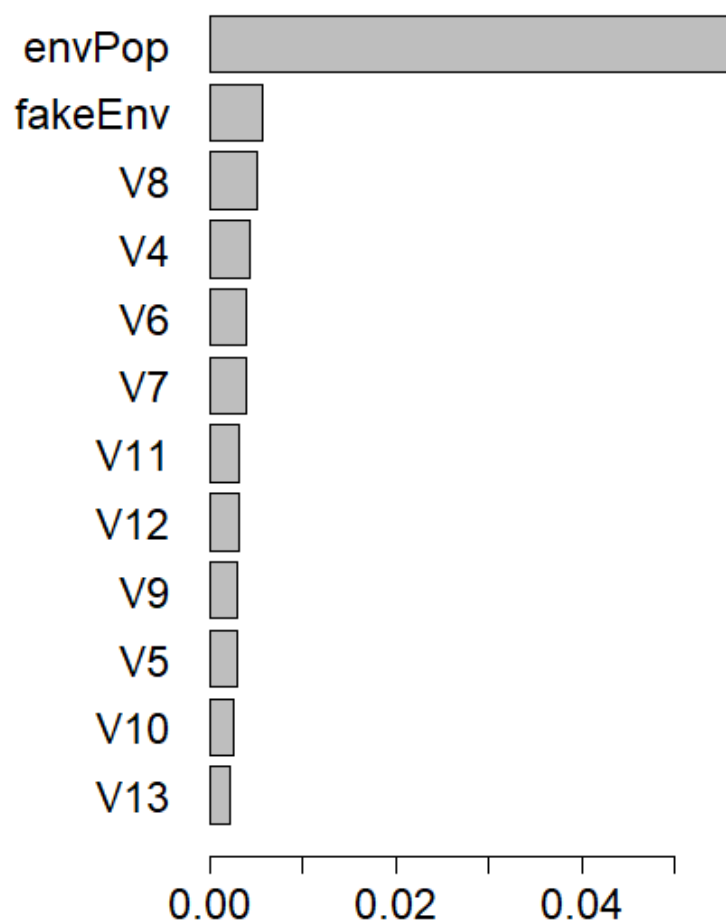

B

 $R^2$  weighted importance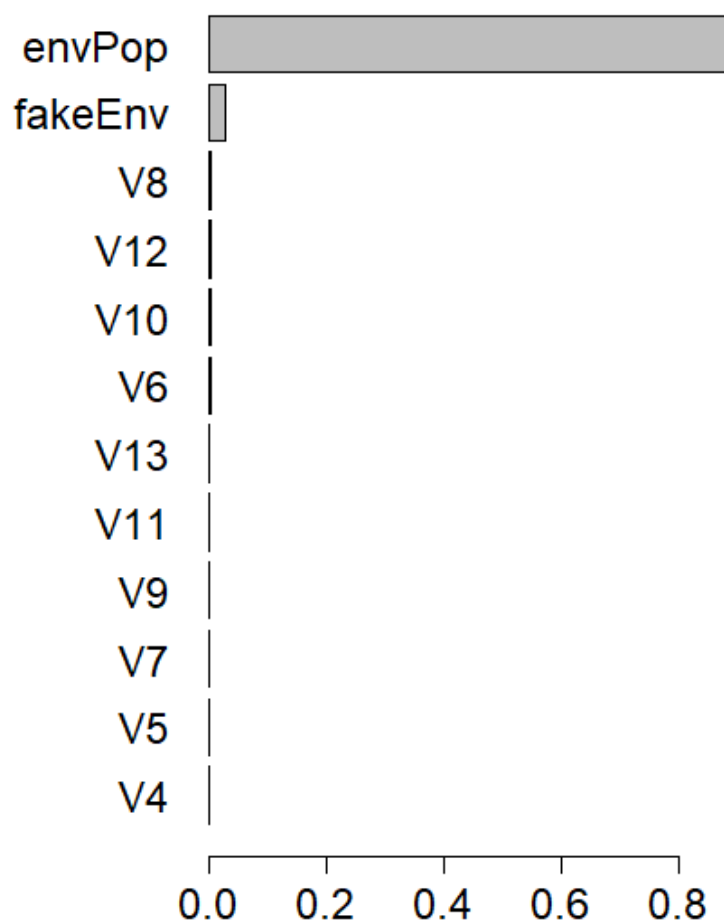

Supplement: Supplementary file 2 — Fig S2 [file EVA-15-403-s001.pdf]

**A**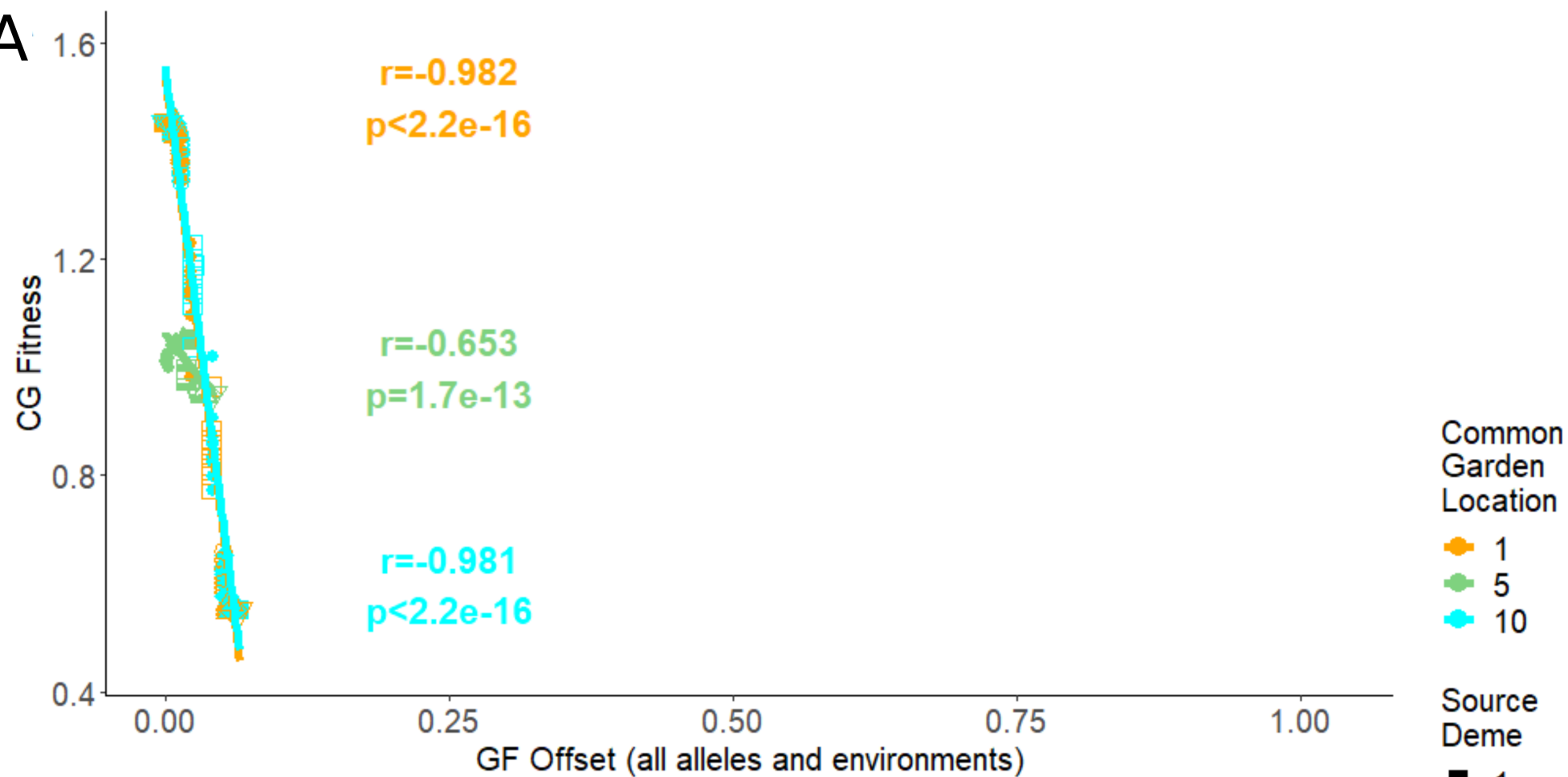**B**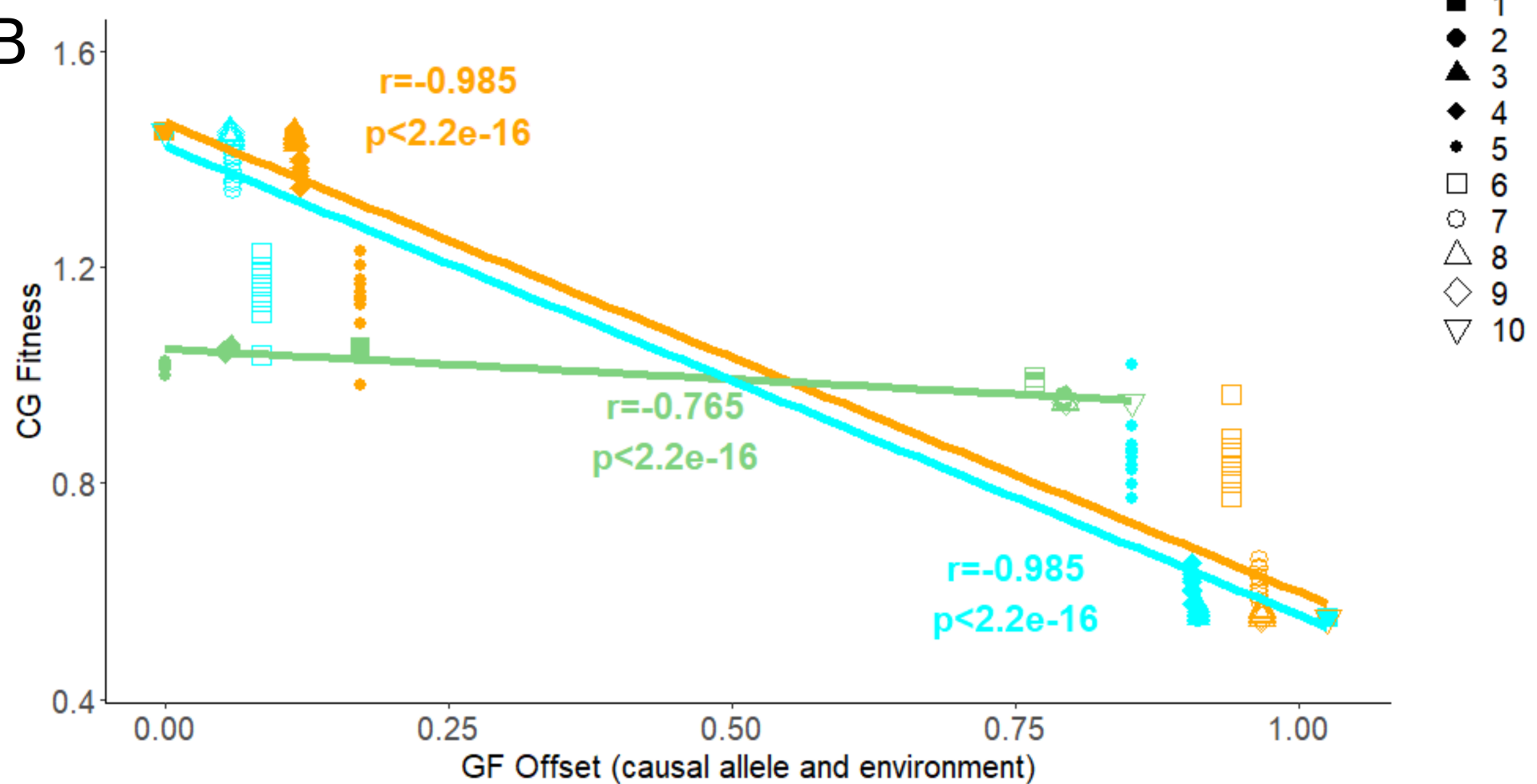

Supplement: Supplementary file 3 — Fig S3 [file EVA-15-403-s003.pdf]

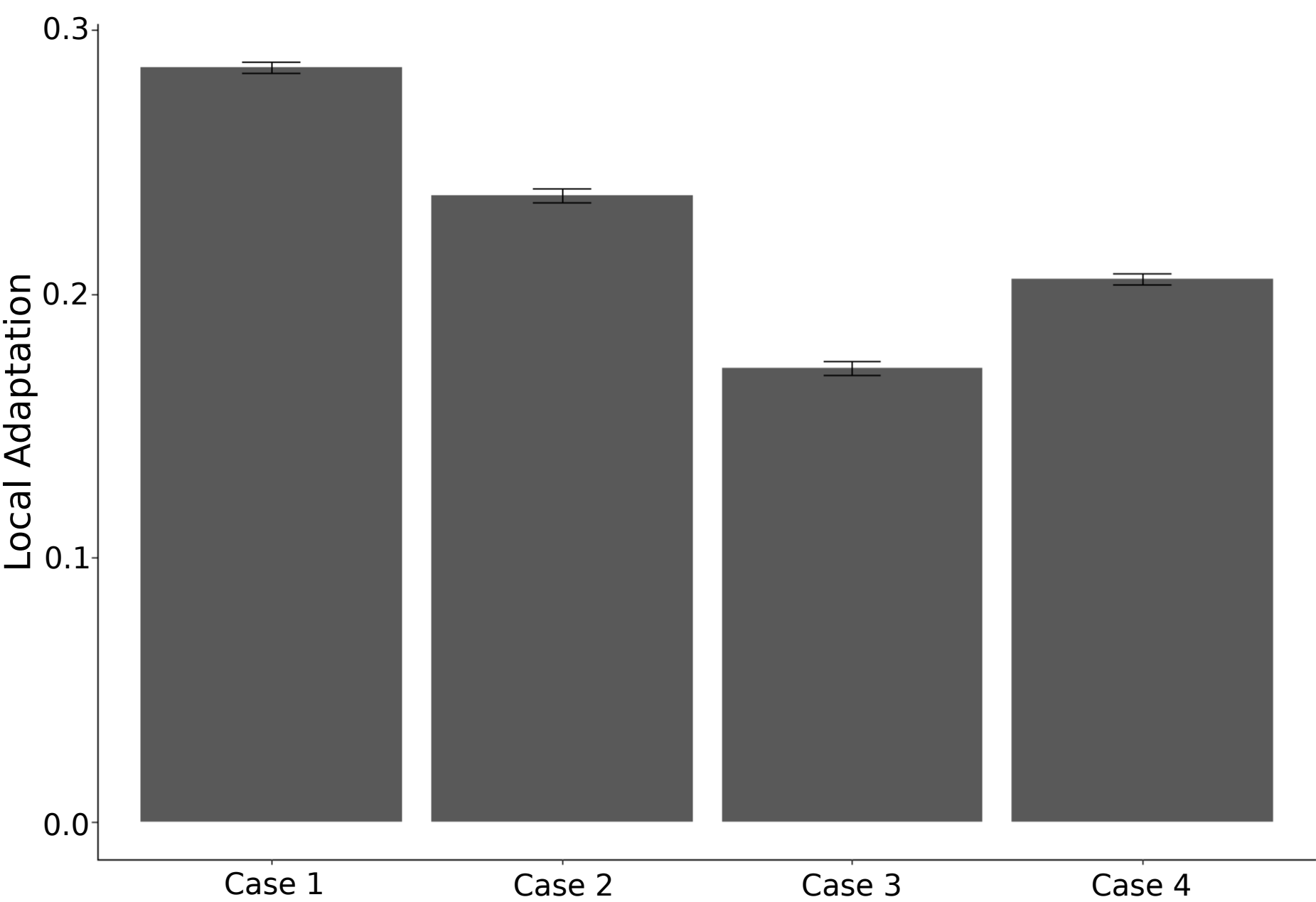

Supplement: Supplementary file 4 — Fig S4 [file EVA-15-403-s008.pdf]

Case 1

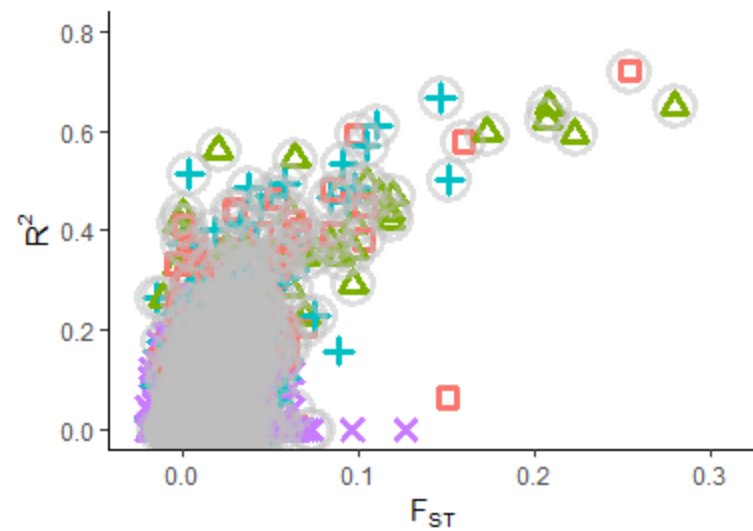

Case 2

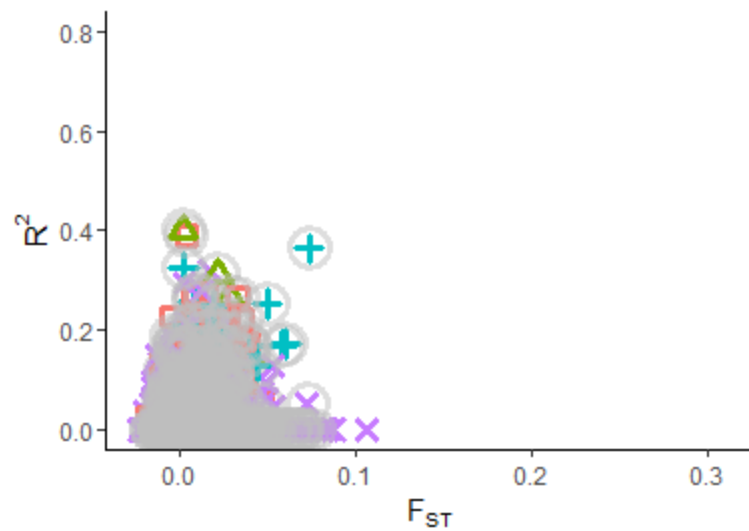Environmental  
Correlation

E1

E1 &amp; E2

E2

Not correlated

Case 3

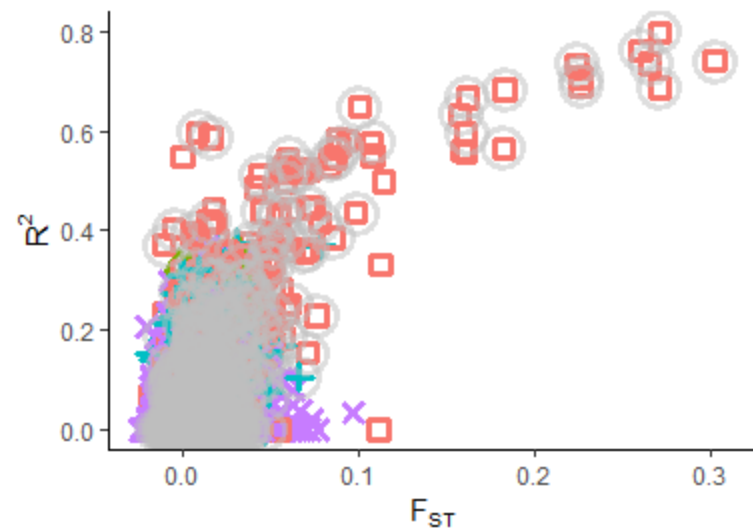

Case 4

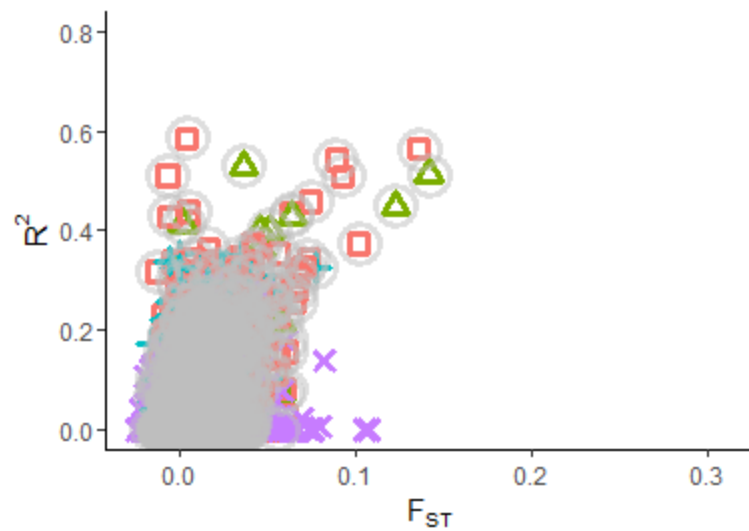

Supplement: Supplementary file 6 — Fig S6 [file EVA-15-403-s005.pdf]
