# Supplementary material for: Seeing the forest for the trees: Assessing genetic offset predictions from gradient forest
Source: Evol Appl. 2022 Feb 25;15(3):403–16. doi: 10.1111/eva.13354 (PMC8965365; doi:10.1111/eva.13354)
Supplement: Supplementary file 5 — Fig S5 [file EVA-15-403-s004.pdf]

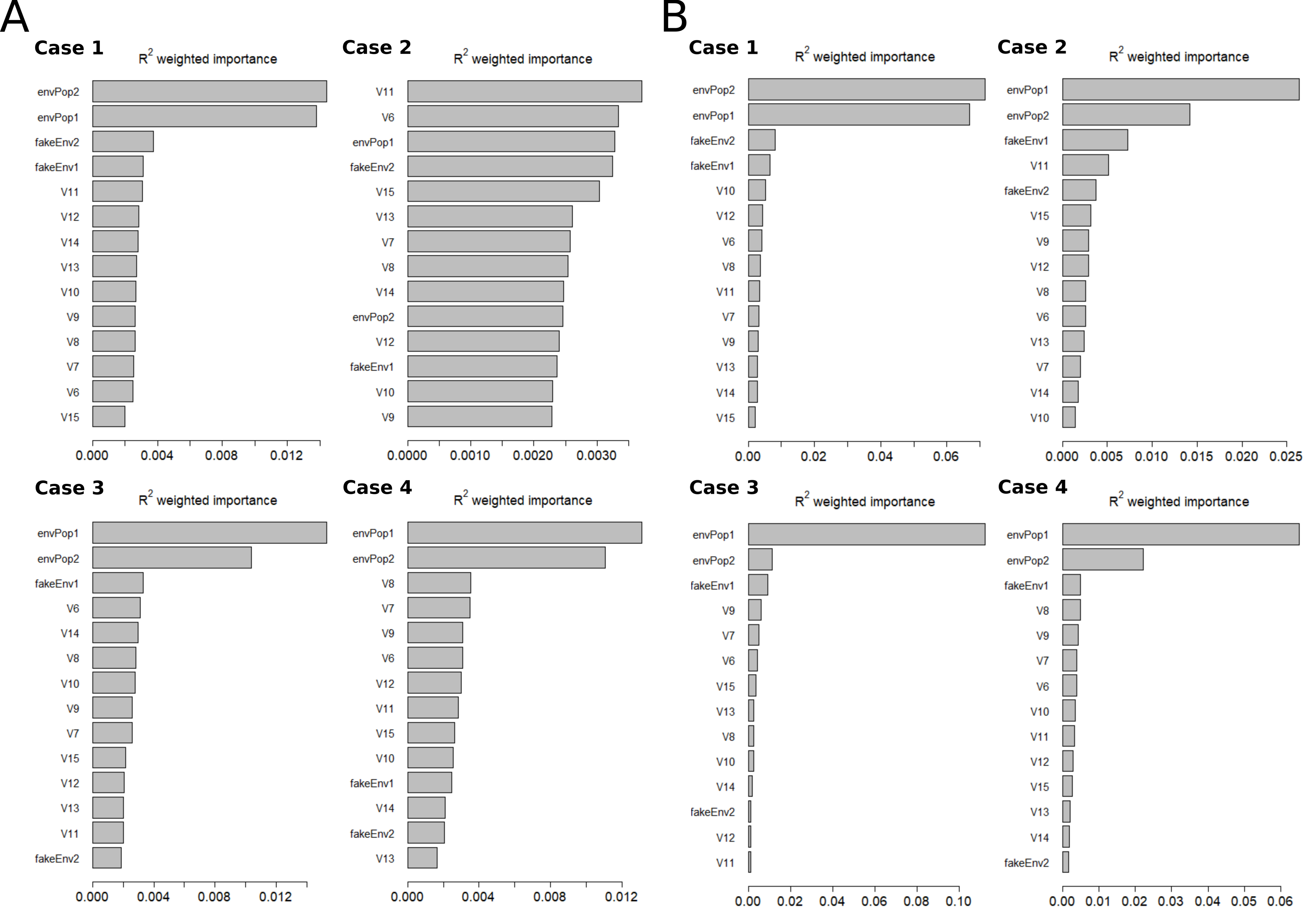

A
Case 1
R2 weighted importance
envPop2
envPop1
fakeEnv2
fakeEnv1
V11
V12
V14
V13
V10
V9
V8
V7
V6
V15
0.000 0.004 0.008 0.012
Case 2
R2 weighted importance
V11
V6
envPop1
fakeEnv2
V15
V13
V7
V8
V14
envPop2
V12
fakeEnv1
V10
V9
0.0000 0.0010 0.0020 0.0030
Case 3
R2 weighted importance
envPop1
envPop2
fakeEnv1
V6
V14
V8
V10
V9
V7
V15
V12
V13
V11
fakeEnv2
0.000 0.004 0.008 0.012
Case 4
R2 weighted importance
envPop1
envPop2
V8
V7
V9
V6
V12
V11
V15
V10
fakeEnv1
V14
fakeEnv2
V13
0.000 0.004 0.008 0.012
B
Case 1
R2 weighted importance
envPop2
envPop1
fakeEnv2
fakeEnv1
V10
V12
V6
V8
V11
V7
V9
V13
V14
V15
0.00 0.02 0.04 0.06
Case 2
R2 weighted importance
envPop1
envPop2
fakeEnv1
V11
fakeEnv2
V15
V9
V12
V8
V6
V13
V7
V14
V10
0.000 0.005 0.010 0.015 0.020 0.025
Case 3
R2 weighted importance
envPop1
envPop2
fakeEnv1
V9
V7
V6
V15
V13
V8
V10
V14
fakeEnv2
V12
V11
0.00 0.02 0.04 0.06 0.08 0.10
Case 4
R2 weighted importance
envPop1
envPop2
fakeEnv1
V8
V9
V7
V6
V10
V11
V12
V15
V13
V14
fakeEnv2
0.00 0.01 0.02 0.03 0.04 0.05 0.06
